# Bioinformatic analysis of fruit-specific expressed sequence tag libraries of *Diospyros kaki* Thunb.: view at the transcriptome at different developmental stages

**DOI:** 10.1007/s13205-011-0005-9

**Published:** 2011-04-21

**Authors:** Gaurav Sablok, Chun Luo, Wan Sin Lee, Farzana Rahman, Tatiana V. Tatarinova, Jennifer Ann Harikrishna, Zhengrong Luo

**Affiliations:** 1 Key Laboratory of Horticultural Plant Biology (MOE), Huazhong Agricultural University, Shizishan, Wuhan, 430070 China; 2 Centre for Research in Biotechnology for Agriculture (CEBAR) and Genetics and Molecular Biology, Institute of Biological Sciences, Faculty of Science, University of Malaya, 50603 Kuala Lumpur, Malaysia; 3 Division of Mathematics and Statistics, University of Glamorgan, Pontypridd, CF37 1DL UK

**Keywords:** *Diospyros kaki*, Expressed sequence tag, GC_3_ biology, MicroRNA, SSRs, SSR-FDM, SNPs

## Abstract

We present here a systematic analysis of the *Diospyros kaki* expressed sequence tags (ESTs) generated from development stage-specific libraries. A total of 2,529 putative tentative unigenes were identified in the MF library whereas the OYF library displayed 3,775 tentative unigenes. Among the two cDNA libraries, 325 EST-Simple sequence repeats (SSRs) in 296 putative unigenes were detected in the MF library showing an occurrence of 11.7% with a frequency of 1 SSR/3.16 kb whereas the OYF library had an EST-SSRs occurrence of 10.8% with 407 EST-SSRs in the 352 putative unigenes with a frequency of 1 SSR/2.92 kb. We observed a higher frequency of SNPs and indels in the OYF library (20.94 SNPs/indels per 100 bp) in comparison to MF library showed a relatively lower frequency (0.74 SNPs/indels per 100 bp). A combined homology and secondary structure analysis approach identified a potential miRNA precursor, an ortholog of miR159, and potential miR159 targets, in the development-specific ESTs of *D. kaki.*

**Electronic supplementary material:**

The online version of this article (doi:10.1007/s13205-011-0005-9) contains supplementary material, which is available to authorized users.

## Introduction

The genus *Diospyros* (Ebenaceae) is a widely distributed heterozygous genus in tropic and subtropic areas of Asia, Africa, and America (Central and North) with complex ploidy levels ranging from diploid (2*n* = 2*x* = 30) to nanoploid (2*n* = 9*x* = 135) (Yonemori et al. 2000). *Diospyros kaki* Thunb. (classified by a prominent Swedish naturalist Carl Peter Thunberg) or Japanese persimmon is the most economically important climacteric fruit species (having varied levels of ploidy; 2*n*, 6*n* and 9*n*) within this genus. In 2007, gross production of persimmon was estimated to be about 2,340,000 tons, of which 89.8% was produced in China, one of the origins of Japanese persimmon (Wang et al. 1997; FAO 2008). Vitamins A and C constitute the major portion of the vitamins present in fresh persimmon fruit.

On the basis of proanthocyanidins (PAs) (colorless phenolic polymers known as useful agents for human health, which show brown coloration upon oxidation), persimmon are further classified into astringent (A)-type fruits and non-astringent (NA)-type fruits (Dixon 2005; Ikegami et al. 2009). A comparative analysis of catechin composition among the five Japanese persimmon demonstrated that epigallocatechin (EGC) is relatively lower in the non-astringent type persimmon (Suzuki et al. 2005). An *AST/ast* allele having allelotypes as astringent (A) and non-astringent (NA) controls the expression of the trait. Expression of homozygous recessive ast allelotype at the AST locus results in the non-astringent (NA) genotype (Kanzaki et al. 2001; Yamada and Sato 2002). Recently, Ikegami and her team (2007) isolated seven genes (*PAL, C4H, CHI, F3H, F30H, ANS,* and *ANR*) from an astringent-type cultivar using suppression subtractive hybridization. Transcription of *PAL, C4H, 4CL, CHS, CHI, F3H, F30H, F3050H, DFR,* and *ANR* genes is high until mid-August, and then declines in October in the astringent-type cultivar (Ikegami et al. 2005a, b). Pang et al. (2007) identified ethylene receptor genes (*DkERS1, DkETR1,* and *DkETR2*) homologous to *Arabidopsis* ethylene receptor genes (ERS1, ETR1, and ETR2) in *D. kaki*. A Myb transcription factor (*DkMyb4*) controls proanthocyanidin biosynthesis in persimmon fruit (Akagi et al. 2009). Overall, *D. kaki* has great potential for becoming a model for understanding important traits in tannins and flavonoid biosynthesis as a fruiting crop species.

*Diospyros kaki* fruiting genotypes have wide morphological, physiological, and molecular diversity. Dominant and transposon-based markers have been described for *Diospyros*, including RAPD, RFLP, IRAP, REMAP, SSAP, SRAP, and AFLP (Yamagishi et al. 2005; Guo and Luo 2006; Du et al. 2009a, b).

In recent years, using in silico approach through mining expressed sequence tags (ESTs) have become an effective way for developing molecular markers such as Simple sequence repeats (SSRs), SNPs, SSR-FDMs for developing the saturated genetic linkage maps for various plant species (Hytena et al. 2010). In addition, the markers so developed not only exhibit higher level of intragenic transferability but also transferability to other closely related genera and may serve as potential markers for species discrimination, evolutionary inference and comparative genomics (Varshney et al. 2005). Extensive analysis has been done using ESTs available in the publicly available databases to identify genes temporally or spatially regulated during fruit growth and development in tomato, grape and apple (Fei et al. 2004; Da Silva et al. 2005; Park et al. 2006).

One of the most recently discovered regulatory mechanisms is post-transcriptional and involves 21–24-nt small RNA molecules (sRNAs). Micro RNAs (miRNAs) are non-protein coding, genomic derived small RNAs that participate in regulation of gene expression at a post-transcriptional level. In plants, they are involved in development, responses to biotic and abiotic stress and whilst some appear unique to a species, a large number of miRNA families are highly conserved across a wide range of plant species (Yang et al. 2007; Jian et al. 2010). miRNA transcripts are capped, spliced, polyadenylated and folded into long hairpin stem-loop precursor molecules (pre-miRNA), which are then processed by RNase III enzymes (Dicer-like in plants; *DCL1*) to form shorter hairpin primary miRNAs (Zhang et al. 2006). A 21–23 base pair double-stranded miRNA: miRNA* duplex is produced by further action of the Dicer enzyme and transported to the cytoplasm where the single-stranded mature miRNA is used as a template for target mRNAs silencing with complementary sequences by cleavage or translational inhibition by an RNA-induced silencing complex (RISC) (Bartel 2004; Zhang et al. 2006). The high sequence conservation of mature plant miRNAs has led to their successful prediction from sequence data using homology-based approaches (Zhang et al. 2005; Sunkar and Jagadeeswaran 2008).

We report here comparative mining of fruit cDNA libraries from different developmental stages of *D. kaki*. We have identified 506 SSRs primer pairs that can be further utilized for the inference of genetic diversity, species discrimination and studying the phylogeography of *Diospyros* genus and in particular *D. kaki*, a potential ortholog of miRNA159 in OYF library, which correlates the potential involvement of miR 159 family in development and relative distribution of SNP and SSR-FDMs markers.

## Materials and methods

### Sequence source and assembly

*Diospyros kaki* ESTs sequences were downloaded from GenBank (dbEST http://www.ncbi.nlm.nih.gov/dbEST) to give a total of 5,053 *D. kaki* ESTs from OYF library and 4,404 *D. kaki* ESTs from MF library. Mature miRNA sequences for all plant species were retrieved from the miRBase Registry (Release 14, September 2009, http://microrna.sanger.ac.uk/) and were used to generate a non-redundant reference set of 1,064 mature miRNA sequences. EST sequences were clustered using CAP3 program to prepare a tentatively consensus (TC) set (Huang and Madan 1999). To compare the relative richness of gene diversity sampled from each library, library-specific contigs, and singletons were compared.

### SSR identification

The identification of SSR containing ESTs was carried out using in-house written program in C, which gives perfect as well as compound SSRs. Repeat patterns ranging from mono- to hexa-nucleotide were identified and systematically analyzed. The parameters defined for the identification of simple sequence repeats were seven minimal repeats for di-, five minimal repeats for tri-, four minimal repeats for tetra- and penta-, and three minimal repeats for hexa-nucleotide. The minimal length of mononucleotide simple sequence repeat was fixed at 14 bp. The poly A and poly T repeats were not considered as SSRs as they exemplify the 3′ end of mRNA/cDNA sequences, thus they were removed. Compound microsatellites were defined as repeats interrupted by a non-repetitive stretch of a maximum of 100 nucleotides.

### SNP identification

Expressed sequence tags sequences were trimmed and a redundancy-based method for SNP confidence measurement, combined with SNP co-segregation (an independent confidence measure) was used to mine SNPs (Barker et al. 2003). The co-segregation score is a measure of whether a predicted SNP contributes to the definition of haplotype. The transition (*T*_s_) versus transversion (*T*_v_) ratio was also calculated for both the libraries to find the DNA substitution dynamics in the *D. kaki* genome.

### Locus-specific primer designing and prediction of SSRs in open reading frames to identify relative biasing

Primer 3 software was being used to design a pair of primers flanking each SSR. The following parameter were used while designing the SSRs primers—optimum primer size was set to 20 where the range was between 18 and 27, optimum annealing temperature was set to 60.0 (the range was between 57.0 and 63.0), and the range of GC content was 20–80% (Shanker et al. 2007). Custom scripts and the standard genetic codes were applied to predict ORFs for all SSR-ESTs. SSR-ESTs were translated in all six ORFs and the longest fragments uninterrupted by stop codons were taken as the putative encoding segment (ORF) of the query SSR-ESTs sequences.

### Annotation of SSR containing sequences, GC_3_ biology and gene ontology

Functional annotations of the SSR-ESTs sequences were determined on the basis of similarity using BLASTX program, available at NCBI (http://www.ncbi.nlm.nih.gov/blast) against non-redundant (nr) protein database entries and the best matches (*E* value <10^−10^) were compared to terms of the Gene Ontology (GO) Consortium (The Gene Ontology Consortium 2000). The resulting proteins obtained through similarity search by BLASTX were allotted to their respective classes. Using GO/UniProt comparison tables, candidate GO assignments were predicted on the basis of EST matches to the UniProt reference sequences.

Coding sequences of four additional Ericales species, such as: *Actinida deliciosa, A. chinesis, Vaccinium corymbosum* and *Camellia sinensis* were obtained from NCBI; in-house C++ code was used to compute position-specific nucleotide composition. In case of *D. kaki*, open reading frames and corresponding proteins were predicted using the assembled contigs of ESTs and nucleotide composition and sequence length was computed for each of the two EST libraries separately.

Using Gene Ontology (The Gene Ontology Consortium 2000) annotation of *Arabidopsis thaliana* (available at http://www.arabidopsis.org), all *D. kaki* protein sequences were aligned to *A. thaliana* using NCBI blastp with *E* value cut-off of 10^−30^, and the GO annotation of the best hit was used to annotate *D. kaki* genes. Chi-squared test (*α* = 0.05) was used to identify significant enrichment of different GO categories in high- and low-GC_3_ genes (Tatarinova et al. 2010)**.** Categories were assigned on the basis of biological, functional, and molecular annotations available from the GO website (http://www.geneontology.org/).

### Identification of functional domains markers (SSR-FDMs)

Using a python script sequences were translated into all six reading frames. In addition, Inter pro scan tool was used to analyze protein domain maintaining default parameter value (Quevillon et al. 2005; Yu et al. 2010). The sequences that contained both SSRs and functional protein domains were selected as SSR-FDMs; however, absence of predicted protein (as non-functional domain) caused exclusion for the sequences from further analysis.

### Homology search and secondary structure prediction for miRNA identification

Candidate miRNA precursor sequences within the EST data were identified using BLAST and MFOLD RNA folding algorithms with parameters described elsewhere (Nasaruddin et al. 2007). Briefly, standalone BLAST (ver. BLAST-2.2.16) was used for local alignment of the EST against the non-redundant query set of 1,064 plant mature miRNA sequences. Default settings were as described elsewhere (Zhang et al. 2005). ESTs sharing homology with miRNAs in the reference set were defined as those containing a predicted mature miRNA with less than four (<4) mismatches compared to a known mature miRNA sequence in the reference set. Putative miRNA orthologs were analyzed using MFOLD RNA folding program (http://mfold.bioinfo.rpi.edu/cgi-bin/rna-form1.cgi) and candidate precursor miRNA (pre-miRNA) were filtered using the characteristics described elsewhere (Zuker 2003; Nasaruddin et al. 2007; Qiu et al. 2007; Xie et al.^2007^). Briefly, (1) the composition of the RNA sequences needs to be folded into a hairpin structure as per the stem-loop precursors. According to this process, each arm of the hairpin will contain ~22 nt mature miRNA sequences; (2) the lower minimal-free energy (MFE) and minimal-free energy index (MFEI) should be compulsorily present in the predicted secondary structure of the miRNA precursors than the tRNA or rRNA; (3) 30–70% of A + U content should be present in the predicted mature miRNA; (4) the mature miRNA sequence is the integral part of the hairpin loop segment. This mature miRNA should have less than six mismatches to the opposite miRNA* sequence of the other arm; (5) any part of mature miRNA:RNA*dimer loop or bulge should contain three nucleotides (maximum). This nucleotides should not be involved in canonical base pairing.

### Prediction of miRNA targets

Potential targets of strong candidate miRNA from *D. kaki* EST were anticipated using RNA hybrid (http://bibiserv.techfak.uni-bielefeld.de/rnahybrid/) (Rehmsmeier et al. 2004). The mature miRNA sequence was used to query the complete EST dataset using the following parameters: helix constraint (−*f*) of 8–12; maximum internal loop size (−*u*) of one and maximum bulge loop size (−*v*) of one (Rehmsmeier 2006). Good candidates were considered those with a negative folding free energy (MFE; ∆Kcal/mol) value below 70% of the MFE value for perfect complementarily and with end overhangs of no more than two nucleotides (Alves et al. 2009). Function of ESTs were predicted using BLASTX program by comparing the sequences against the non-redundant NCBI protein database with a cut off*E* value of 10^−4^ and 40% minimum identity score.

## Results and discussion

### Sequence assembly

Expressed sequence tags, represent partial and redundant cDNA sequences, making it difficult to analyze them effectively for putative mining of markers. To construct longer and less redundant sequence sets, we assembled ESTs by library, using CAP3 (Huang and Madan 1999). In OYF library, clustering of 5,053 sequences yielded 658 tentative consensus (TC) sequences with 3,117 sequences remained unclustered. In MF library, clustering of 4,404 sequences yielded 604 tentative consensus (TC) sequences with 1,925 sequences as singletons. The average length of the tentative consensus (TC) was 521 and 368 bp in OYF and MF library respectively. The diversity in ESTs libraries was confirmed by diversity index depicting higher degree of transcript diversity (Table 1).

**Table 1:** Summary of in silico mining of *Diospyroskaki* cDNA libraries for assembly and repeat analysis

Parameters	Values	
	OYF Library	MF Library
Total number of EST	5,053	4,404
Total number of contigs	658	604
Total Number of ESTs left to assemble	3,117	1,925
Redundancy index (%)	38.31	56.28
Total number of unigenes sequences searched	3,775	2,529
Total number of SSRs after removing poly A and poly T	407	325
Average UniGene length including poly A and poly T	521.12	367.56
Diversity index (%)	75	57.4
Repeat type		
Mono-nucleotide	3 (0.73)*	4 (1.23)
Di-nucleotide	229 (56.3)	175 (53.84)
Tri-nucleotide	101 (24.81)	98 (30.2)
Tetra-nucleotide	18 (4.42)	20 (6.2)
Penta-nucleotide	4 (1.0)	6 (1.8)
Hexa-nucleotide	52 (12.8)	22 (6.8)

* Data in parentheses is the percentage value of the repeat type

### Screening, frequencies, primer designing and annotation of *D. kaki* SSRs-ESTs

**Fig. 1:**
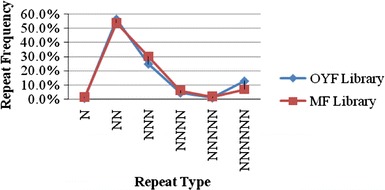
Frequency distribution of library specific repeat types identified in *Diospyros kaki*

In the present study, library-specific tentative consensus (TC) set of *D. kaki* were mined for SSRs with a minimum length of 14 bp. A total of 407 and 325 SSRs were detected in the OYF and MF libraries respectively; excluding poly A and poly T. Poly (A/T) were excluded (Karaoglu et al. 2004). In the OYF library, 5,053 sequences represent 407 SSRs with an average density of one SSR per 2.92 kb whereas in MF library from a number of 4,404 sequences screened only 325 SSRs were detected demonstrating average density of one SSR per 3.16 kb. The frequencies of SSRs with mono-, di-, tri-, tetra-, penta- and hexanucleotide repeat units are shown in Table 1. The most frequent repeat type found among different developmental libraries analyzed were di-nucleotide repeats (53.8%; 53.6%) followed by tri-nucleotide (30.2%; 24.8%), hexa-nucleotide (6.8%; 12.8%), tetra-nucleotide (6.2%; 4.4%), penta-nucleotide (1.8%; 1.0%), and mono-nucleotide repeats (1.2%; 0.7%), respectively (Fig. 1).

**Fig. 2:**
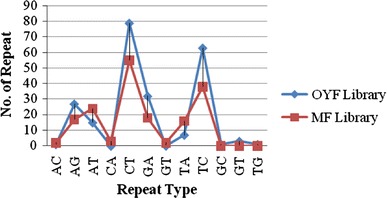
Frequency distribution of library specific dinucleotide repeat types identified in *Diospyros kaki*

**Fig. 3:**
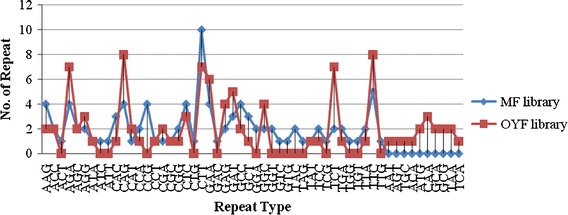
Frequency distribution of library specific trinucleotide repeat types identified in *Diospyros kaki*

**Fig. 4:**
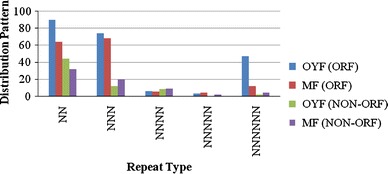
Relative distribution patterns of SSRs with primer pairs in ORF and Non-ORF among di-, tri-, tetra-, penta- and hexa-nucleotide repeat in OYF and MF library

In both cDNA libraries surveyed, the mono-nucleotide repeats were relatively low when compared with other repeats. We further analyzed the observed abundant dinucleotide and trinucleotide repeat patterns (Figs. 2, 3) and reduction in the frequency of SSRs before and after assembly is both the libraries **(**Table 2). Similar patterns have been observed in the mining of the EST-SSRs markers in cereal species (Varshney et al. 2002). In case of the OYF library*,* out of 407 SSRs detected, primers could be designed only for 286 (70.2%) SSRs, whereas for the MF library*,* out of 325 SSRs detected, primers could be designed only for 220 (67.6%) SSRs (Supplementary Table 1). SSRs with primer pairs with respect to ORF were predicted in both MF and OYF libraries. In OYF library, out of 407 SSRs identified, 220 SSRs with primer pairs (54.0%) were found with respect to ORF. In the MF library, out of 325 SSRs identified, 153 (47.0%) SSRs with primer pairs were found with respect to ORF (Fig. 4).

**Table 2:** Frequency of SSRs in the EST sets before and after assembly

Source of ESTs	Before assembly			After assembly		
	No. of sequence	No. of SSR-ESTs (1–6 bp)	SSR-ESTs (%)	No. of unigenes	No. of SSR-ESTs (1–6 bp)	SSR-ESTs (%)
OYF library	5,053	586	11.5	3,775	407	10.78
MF library	4,404	611	13.8	2,529	325	12.8

Genic as well as the intergenic regions displayed the presence and absence of SSRs (Katti et al. 2001). A higher percentage of dinucleotide repeats in non-ORF regions may reflect natural evolution to maintain the conservation of functionality of all genes and their products (Fig. 4). Nevertheless, recent studies indicate that SSR expansions and/or contractions in protein-coding regions may cause a gain or loss of gene function through frame shift mutations (Fondon et al. 2008).

Simple sequence repeat-containing sequences (SSRs-ESTs) were annotated against the non-redundant (nr) protein database available at http://www.ncbi.nlm.nih.gov. Out of 296 MF library-derived SSRs-ESTs, 131 were found to homologous (44.3%) whilst for the OYF library, out of 352 SSRs-ESTs, homologs were available for only 146 (41.5%) sequences.

### SNP identification

Redundancy-based SNPs mining resulted in identification of 68,067 SNPs and 4,273 indels in the *D. kaki* transcriptome. SNPs occurred at a frequency of one out of every 10 bp and indels at one in every 152 bp. A total of 28,232 transitions and 39,835 transversions were reported in this study. For explaining the nucleotide substitution dynamics, transition (*T*_s_) to transversion (*T*_v_) ratio was calculated because it provides insights into the process of molecular evolution. The transition/transversion ratio is relatively low in the OYF library (0.69) compared to the MF library (1.39) the overall transition (*T*_s_) to transversion (*T*_v_) ratio is 0.70, which indicates an relative increase of transversion (*T*_v_) over transitions (*T*_s_) (Table 3).

**Table 3:** SNP analysis

S. No.	Parameter	OYF Library	MF library	Total
1	Total sequences analyzed	1,936	2,479	4,415
2	Total number of TC sequences	658	604	1,262
3	Total SNP and indels detected	70,008	2,332	72,340
4	Total consensus size (bp)	334,271	314,897	649,168
5	Total transitions	27,016	1,216	28,232
6	Total transversions	38,966	869	39,835
7	Total indel	4,026	247	4,273
8	*T*_s_/*T*_v_	0.69	1.39	0.7
9	SNP frequency	1SNP/5 bp	1SNP/151 bp	1SNP/10 bp
10	Indel frequency	1Indel/83 bp	1Indel/1,274 bp	1Indel/152 bp

Earlier studies have demonstrated higher rate of transitions over transversions due to abundant hypermutable methylated dinucleotides (5′-CpG-3′) (Ching et al. 2002; Strandberg and Salter 2004; Newcomb et al. 2006). However, in the present study, transversions prevail over the transitions. Neighbouring nucleotide effect demonstrates that the probability of transversion increases when the number of purines increases at the immediate adjacent sites (Zhongming and Boerwinkle 2002). Similar patterns of transversions over transitions were observed for genes on rice chromosome 8 (Wu et al. 2004).

In plant chloroplasts, an increase in transversions with increase in the A + T content of adjacent nucleotides has been observed (Morton 1995). These studies illustrate that the transition bias is not universal and supports the findings of the present study. However, the frequency of SNPs detected in this study is higher than the frequency of EST derived SNPs generally reported in earlier studies; 1 SNP/61 bp in *Zea mays*, 1 SNP/540 bp in *Triticum aestivum*, 1 SNP/123 bp in *Sorghum bicolor* and1 SNP/58 bp in *Secale cereale* transcriptome (Ching et al. 2002; Somers et al. 2003; Hamblin et al. 2004; Varshney et al. 2007). Possible reasons for variation in SNP density may perhaps be due to dissimilarity in the quantity of data analyzed.

### Functional domains markers (SSR-FDMs)

Tentative consensus (TC) from the respective libraries was analyzed for functional domain markers excluding the mononucleotide repeats from this analysis. The translation of the sequences was performed in all six reading frames. InterProScan tool was used to analyse the resulting amino acid sequences from the longest reading frame (http://www.ebi.ac.uk/Tools/pfa/iprscan/). In the case of the OYF library, four potential SSR-FDMs were observed and Vps4 oligomerisation domain and the C2 calcium/lipid-binding domain were identified as major functional domains. The MF library displayed 10 potential SSR-FDMs but Basic-leucine zipper (bZIP) transcription factor, Glycoside hydrolase were observed as major functional domains. Therefore, this strategy not only implicates the evaluation of SSR polymorphisms, but also predicts function viability of these marker sequences. Association between candidate functional markers and trait of interest can be investigated by mapping SSR-FDMs.

### miRNA and miRNA target identification

**Fig. 5:**
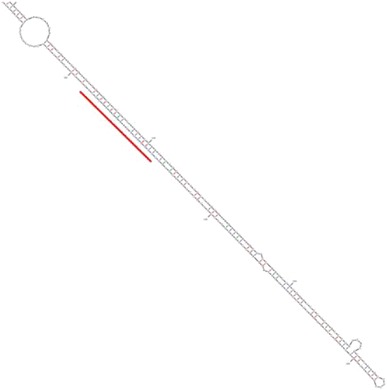
The predicted secondary structure of candidate miR159 precursor from *Diospyros kaki*. The secondary structure for EST DC584412.1 (candidate miR159) predicted using MFOLD (Zuker 2003). The sequence encoding the predicted mature miR159 is indicated by the line below the strand in which the mature miRNA is located

After removal of redundant EST sequences, a total of six ESTs from the MF library and seven from the OYF library were found to align with a known mature miRNA from the plant reference set with fewer than 4 mismatches within the mature miRNA sequence (Table 4). Of these candidates, one EST fulfilled the criteria for miRNA precursors based on the MFE (−137.40 kcal/mol) and secondary structure as predicted by MFOLD RNA folding program (Zuker 2003). A potential ortholog of miR159 was identified from OYF library (Fig. 5)*.* A discovery rate of one miRNA precursor from a set of 9,457 ESTs of *D. kaki* lies within the expected range as per previous reports, which ranges from 0.83 per 10000 EST reported for *Malus domestica* to 1.69 per 100,00 EST from *Gossypium hirsutum* (Qiu et al. 2007; Gleave et al. 2008).

**Table 4:** ESTs from Libraries for ovary and young fruit and for mature fruit less than four mismatches to mature miRNA from rice or from *Arabidopsis*

S. No.	miR Family^a^	Length of mature miRNA	Match (BLAST)	EST
1	osa-miR414	21	18/21	DC588681.1^b^
2	ath-miR414	21	18/18	DC591906.1^b^
3	osa-miR395o	21	20/21	DC591801.1^b^
4	peu-miR2914	23	22/22	DC592202.1^b^
5	ppt-miR1038-3p	21	18/20	DC589073.1^b^
6	peu-miR2910	21	21/21	DC589557.1^b^
7	osa-miR159a.1; osa-miR159b	21	21/21	DC584412.1
8	sof-miR408e	21	19/20	DC585074.1
9	osa-miR408	21	18/18	DC584676.1
10	osa-miR414	21	18/20	DC588395.1
11	ath-miR414	21	19/20	DC588139.1; DC586156.1

^a^Identifiers from miRBase Registry. Only the highest scoring match is shown

^b^Identifiers for EST from the Mature Fruit library

Possible targets of the potential *D. kaki* miR159 (EST DC584412) were identified using RNA hybrid (Table 4). The candidates were screened and those having an MFE of −29.7 kcal/mol or lower (i.e. a minimum of 70% of the MFE for a perfect match) and two or fewer nucleotide overhangs at either end of the duplex were selected. A perfect match for the mature miRNA159 sequence has a predicted MFE value of −42.4 kcal/mol using RNA hybrid. Whilst six ESTs were identified as potential targets of predicted *D. kaki* miR159, only one of these (EST DC590670) matched an identified protein glutathione S-transferase (Table 5).

**Table 5:**
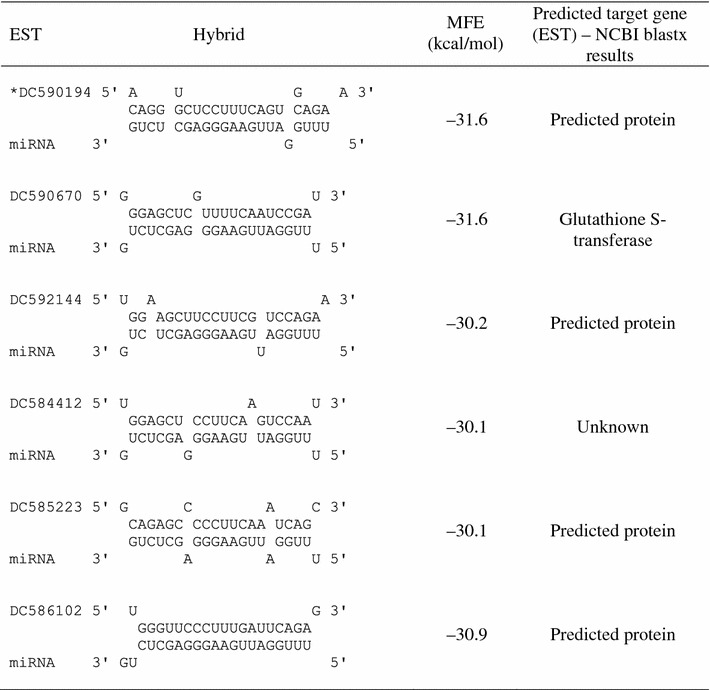
Predicted miRNA159 targets and functions

* ESTs DC584412, DC585223 and DC586102 OYF library, whilst DC590194, DC590670 and DC592144 were from the MF library

Previous reports show that miR159 targets include MYB transcription factors (Jones-Rhoades et al. 2006; Mallory and Vaucheret 2006), however, more recent studies suggest that, along with other miRNA families that are highly conserved across plant species, targets of miR159 are involved in diverse biological processes including gametogenesis, anther development, gibberellins signaling, and ethylene biosynthesis (Alves et al. 2009). Thus, the six EST identified as possible targets in this study may also represent a similar range of diversity.

### GC_3_ biology

**Fig. 6:**
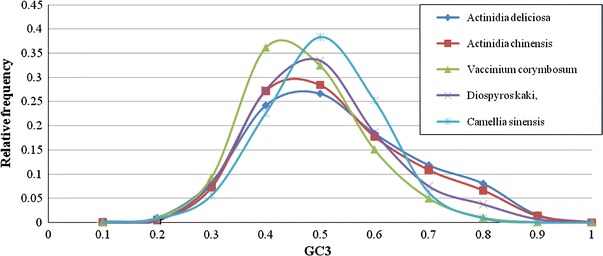
GC3 distribution for selected representatives of the Ericales order

Unimodal GC_3_ profile of *D. kaki* CDS is typical for the Ericales order and other dicot plants (Fig. 6) (Tatarinova et al. 2010). Both ESTs libraries (OYF and MF) have similar GC content (Table 6). However, the contigs assembled for the mature fruit ESTs library are, on average, approximately 100 nucleotides longer. In order to analyze dependence between GC_3_ and GO, we took 10% of highest and lowest genes by GC_3_ in *D. kaki* and four other Ericales genomes (*A. chinesis*, *Actinidia delicosa*, *V. corymbosum* and *C. sinesis*). According to GO classification, high GC_3_ genes are over-represented in stress response genes, kinases, transcription factors and located in apoplast, membranes and cell wall (Table 7). Low GC_3_ genes are over-represented in genes involved in protein and nucleotide binding and located in nucleus, cytosol, and cytoplasm.

**Table 6:** GC variations across the different libraries of *Diospyroskaki*

Library type	Length	GC_1_	GC_2_	GC_3_	GC	N
Ovary and young fruit library	177	0.51	0.45	0.50	0.49	2,322
Mature fruit library	280	0.52	0.42	0.50	0.48	2,228

**Table 7:** GO-term enrichment for high- and low-GC3 *Diospyroskaki* genes

Ontology type	Description	High	Low	Ratio
Molecular function	Structural constituent of ribosome	27	2	13.500
Cellular localization	Ribosome	21	2	10.500
Cellular localization	Apoplast	18	2	9.000
Molecular function	Translation	27	3	9.000
Molecular function	Response to wounding	17	3	5.667
Cellular localization	Endomembrane system	62	13	4.769
Cellular localization	Cell wall	39	9	4.333
Cellular localization	Endoplasmic reticulum	25	8	3.125
Molecular function	Response to salt stress	26	9	2.889
Cellular localization	Membrane	73	26	2.808
Molecular function	Kinase activity	25	9	2.778
Molecular function	Transport	16	6	2.667
Molecular function	Protein serine/threonine kinase activity	16	6	2.667
Molecular function	Transcription factor activity	30	13	2.308
Molecular function	Molecular function	134	59	2.271
Cellular localization	Integral to membrane	18	8	2.250
Molecular function	Protein amino acid phosphorylation	19	9	2.111
Biological process	Biological process	155	75	2.067
Cellular localization	Nucleolus	18	9	2.000
Cellular localization	Plant-type cell wall	17	9	1.889
Cellular localization	Cellular component	138	76	1.816
Cellular localization	Vacuole	41	23	1.783
Molecular function	Defense response	14	8	1.750
Molecular function	Protein binding	41	24	1.708
Cellular localization	Chloroplast stroma	17	10	1.700
Molecular function	ATP binding	22	13	1.692
Cellular localization	Mitochondrion	37	22	1.682
Cellular localization	Chloroplast	97	58	1.672
Molecular function	DNA binding	25	15	1.667
Cellular localization	Chloroplast envelope	16	10	1.600
Molecular function	Metabolic process	20	13	1.538
Cellular localization	Plasma membrane	90	61	1.475
Cellular localization	Nucleus	56	38	1.474
Cellular localization	Cytoplasm	19	13	1.462
Molecular function	Transferase activity, transferring glycosyl groups	12	9	1.333
Molecular function	Response to cadmium ion	16	14	1.143
Molecular Function	Catalytic activity	28	25	1.120
Molecular function	Zinc ion binding	13	13	1.000
Cellular localization	Cytosol	19	21	0.905
Molecular function	ATP binding	26	29	0.897
Molecular Function	Embryonic development ending in seed dormancy	13	15	0.867
Molecular function	Nucleotide binding	13	15	0.867
Molecular function	Nucleic acid binding	12	18	0.667
Molecular function	Protein binding	13	21	0.619
Molecular function	Binding	13	23	0.565

The present study was aimed to generate resources that can be utilized for the identification and characterization of *D. kaki* germplam. The markers identified here can be used for subsequent prediction of germplasm diversity, phylogeography and species discrimination among the *Diospyros* genus.

## Electronic supplementary material

Below is the link to the electronic supplementary material.

Table 1. List of SSRs with primer information (XLS 275 kb)

## Abbreviations

AFLP: Amplified fragment length polymorphism
IRAP: Inter-retrotransposon amplified polymorphism
MF library: Mature fruit library
ORF: Open reading frame
OYF library: Ovary and young fruit library
RAPD: Random amplified polymorphic DNA
RFLP: Restriction fragment length polymorphism
REMAP: Retrotransposon microsatellite amplified polymorphism
SRAP: Sequence-related amplified polymorphism
SNPs: Single nucleotide polymorphisms
SSAP: Sequence-specific amplified polymorphic
SSRs: Simple sequence repeats
SSR-FDMs: Simple sequence repeats-functional domain markers
